# Cross-Country Comparisons of Physical Activity and Sedentary Behavior among 5-Year-Old Children

**DOI:** 10.1155/2020/7912894

**Published:** 2020-06-01

**Authors:** Kerry L. McIver, Russell R. Pate, Marsha Dowda, Suzanne Bennett Johnson, Jimin Yang, Martha Butterworth, Xiang Liu

**Affiliations:** ^1^University of South Carolina, Arnold School of Public Health, Department of Exercise Science, 921 Assembly Street, Columbia, SC 29208, USA; ^2^Florida State University, College of Medicine, Department of Behavioral Sciences and Social Medicine, Tallahassee, FL 32306, USA; ^3^University of South Florida, Health Informatics Institute, Tampa, FL 33620, USA

## Abstract

**Purpose:**

Previous studies have observed that physical activity (PA) levels tend to be lower in the U.S. population than in many other countries. Within the U.S., PA levels in children are lower in the South than in other regions. Cross-country and interregional differences in PA have not been studied in young children.

**Methods:**

In an ongoing study of children at genetic risk for Type 1 diabetes, PA was measured by accelerometry in samples of 5-year-old children (*n* = 2008) from Finland (*n* = 370), Germany (*n* = 85), Sweden (*n* = 706), and the U.S. (*n* = 847). The U.S. sample was drawn from centers in Washington State, Colorado, and Georgia/Florida. Children wore accelerometers for 7 days, and the data were reduced to daily minutes of light-, moderate- (MPA), vigorous- (VPA), and moderate-to-vigorous- (MVPA) intensity PA and sedentary behavior. Multiple regression was used to compare children across countries and across regions in the U.S, adjusting for wear time, body mass index, and demographic characteristics.

**Results:**

After adjusting for previously mentioned factors, MVPA and MPA were lower in U.S. children than those in Finland and Sweden. Estimates of physical activity were higher in Finland than in other countries, although not all comparisons were significantly different. U.S children spent significantly more time in sedentary behavior than children in Finland (*p* < 0.0001). Within the U.S., children's PA was consistently lowest in Georgia/Florida and highest in Washington.

**Conclusions:**

Cross-country differences in PA, previously reported for adults and adolescents, are evident in 5-year-old children. In general, PA levels are lower in U.S. children than their European counterparts, and within the U.S., are lower in Georgia/Florida and Colorado than in Washington. Future studies should be designed to identify the factors that explain these differences.

## 1. Introduction

Physical activity exerts a powerful and beneficial influence on a wide range of health outcomes [1], and these relationships have been demonstrated in youth as well as adults [2]. Among children and adolescents, higher levels of physical activity (PA) are associated with a reduced risk for development of overweight, better cardiometabolic risk status, and improved bone health [1]. Accordingly, public health authorities in the U.S. and many other countries have established surveillance systems to monitor PA in young persons [3] and have adopted promotion of PA in youth as a public health priority [1, 4]. While much of the focus has been on children of school age, 6 to 18 years, assessment and promotion of PA in children of preschool age have become more common in recent years. This trend has been driven, in part, by the observation that rates of overweight and obesity have increased in 3-5-year-old children [5].

Increases in the prevalence of obesity have been observed in most countries in the world, but those increases have been particularly dramatic in North America [6, 7]. In the U.S., for example, obesity rates in 2- to 5-year-old children increased from 7.2% to 9.4% between 1988-1994 and 2013-2014 [5]. In contrast, English 2- to 5-year-olds show lower rates over a similar period of time (3.8% to 6.3%) [8]. The factors that explain cross-country differences in obesity rates have not been fully elucidated, but it is likely that differences in PA behavior may contribute [9, 10]. In a large-scale comparison of obesity rates and PA levels in children across 12 countries, it was observed that PA levels tended to be the lowest in the countries in which obesity rates were the highest [11]. However, children of preschool age were not included in that study. Correlates of PA behavior, however, have been studied in preschool-aged children, including age, gender, race/ethnicity, family socioeconomic status (SES), BMI, and season. Apart from boys being more active than girls, results from previous studies have shown varied and inconclusive findings for associations between demographic, biological, and environmental factors and PA outcomes in this age group [12–14].

TEDDY, The Environmental Determinants of Diabetes in the Young, is an ongoing study seeking to identify the environmental triggers of type 1 diabetes (T1D) in genetically at-risk children [15]. The study is being conducted in a birth cohort recruited in four countries, including the U.S. As part of the comprehensive TEDDY study protocol, PA is being measured in this cohort via accelerometry on an annual basis, beginning when the children are five years of age. These data provide a unique opportunity to compare PA levels of children across countries. Therefore, the purpose of this study was to compare PA levels and sedentary behavior in young children across the four countries included in TEDDY. A secondary purpose was to make similar comparisons across three geographically and culturally distinct U.S. regions.

## 2. Methods

### 2.1. Study Design

A cross-sectional study design was applied using data from The Environmental Determinants of Diabetes in the Young (TEDDY) study. TEDDY is an ongoing prospective cohort study that is examining factors that relate to the development of T1D in a sample of genetically at-risk children from 4 countries (U.S., Finland, Germany, and Sweden). In the U.S., data are being collected from 3 regions, in Colorado, Georgia/Florida (Southeast), and Washington. The details of the TEDDY study design and methodology have been published previously [15, 16]. Briefly, parents of infants at risk for T1D based on genotyping at birth were asked to participate in the study. TEDDY participants are assessed at regular intervals and are being followed from birth to age 15 years or to the development of T1D [15]. Informed consent was obtained from all participants prior to data collection. Data for the present study include demographic information obtained when the child was 9 months of age and PA data measured via accelerometry when the child was 5 years of age. Children who were islet autoantibody positive at or before the 5-year-old visit (indicating that an autoimmune process associated with T1D may have begun) were excluded from analyses (*n* = 183).

### 2.2. Demographic Characteristics, Season, and Body Mass Index (BMI)

At the child's 9-month-old clinic visit, parents reported child demographic characteristics, including gender, race, ethnic minority status, and maternal education. In the U.S., race was reported as Hispanic, White Non-Hispanic, African-American Non-Hispanic, and Other. U.S. participants were classified as “ethnic minority” if “the mother's first language was not English, the mother was not born in the U.S., or the child was identified as a member of an ethnic minority group based on the U.S. Census definition” [17]. For European participants, a child was classified as an ethnic minority if the child's mother's first language or country of birth was other than that of the TEDDY country in which the child resided [17]. Maternal education was reported on a ten-category scale and recoded for this study to represent two categories: less than college graduate and college graduate or higher. Season of the year (spring, summer, fall, and winter) was also determined based on data collection date. Weight and height were measured according to standard protocols using an electronic scale and a wall-mounted stadiometer. For participants unable to attend a clinical visit, height and weight were abstracted from medical records [18]. BMI (kg/m^2^) was calculated from the average height and weight for each participant.

### 2.3. Physical Activity

Physical activity at the child's 5-year-old visit was measured by accelerometry. Participants were given an ActiGraph accelerometer (model GT3X+, Fort Walton Beach, FL) to wear for at least 7 consecutive days. Monitors were worn on elastic belts around the waist, with the device placed on the right hip, during all waking hours, exclusive of water activities. The accelerometers were initialized to collect data at 80 Hz and were downloaded as 1-second epoch data. Using SAS programs, data were reintegrated into 60-second epoch data files and analyzed for activity intensity levels. Periods of ≥60 minutes of consecutive zero counts were defined as nonwear and set to missing. Age-specific cut-points were used to determine the average minutes per day each child spent in light- (101-1290 counts per minute (cpm)), moderate- (MPA; 1291-3580 cpm), vigorous- (VPA; >3581 cpm), and moderate-to-vigorous- (MVPA) intensity PA (>1291 cpm), as well as total (light+MVPA) PA (TPA) and sedentary behavior [19, 20]. Minutes with <100 counts were categorized as sedentary. Days on which a child had 8+ hours of accelerometer wear were considered valid, and children with at least 3 days of compliant wear were included in the analysis.

### 2.4. Statistical Analysis

Demographic variables were summarized (means (SD) for continuous variables and *n* (percent) for categorical variables for each country and region of the U.S. For descriptive variables of interest, analysis of variance (ANOVA) or logistic regression was used to determine if there were differences between the four countries and between the three U.S. regions.

Mixed model regression analyses, adjusted for wear time, were used to determine the influence of demographic factors including age, gender, mother's education, ethnic minority, BMI, and season on estimates of light, MPA, VPA, MVPA, TPA, and sedentary behavior. Two-tailed *p* values less than 0.05 were considered to be statistically significant.

Linear mixed regression models with least square means, which were Bonferroni corrected, were used to determine if intercountry differences existed for each outcome variable of interest: sedentary, light, MVPA, VPA, and TPA min/day, after adjusting first for monitor wear time, and demographic factors including age, gender, mother's education, ethnic minority, season, and BMI. Models were also fit to determine if there were differences in the outcome variables between the U.S. regions after adjustment for wear time, and demographic variables including age, gender, mother's education, race/ethnicity, season, and BMI. All analyses were performed in SAS (9.4).

## 3. Results

Participant characteristics are summarized in Table 1, which provides information for the overall sample as well as the samples for each country and U.S. region. Approximately equal percentages of males and females were included in the overall sample and in each subsample. Children in the total sample were, on average, 61.2 months old and, despite some small though statistically significant differences, this approximate age was consistent across the subsamples. In the U.S., the samples were predominately Non-Hispanic White, though in Colorado, the sample was 27% Hispanic. Among the participating countries, ethnic minority status was most prevalent in the U.S. (26%), and among the U.S. sites, it was highest in Colorado (34%). Mother's education was different across the countries, with more mothers in the U.S. and Finland reporting education levels of college graduate or higher and more mothers in Germany and Switzerland reporting education levels less than college graduates. There were no differences in the percentages of children completing data collection across the seasons of the year. BMI among participants in Germany was lower than that of participants in other counties, and there were no differences in BMI among U.S. participants.

Results from the multiple linear regression analyses for PA and sedentary variables and demographic factors are presented in Table 2. Gender was significantly associated with intensities, with females less active and more sedentary than males. Children who were measured in spring engaged in more minutes of MPA, VPA, MVPA, and TPA and less minutes of sedentary behavior than children measured in winter. Children measured in summer had significantly more minutes of VPA than those measured in winter. Children with a higher BMI engaged in fewer minutes of VPA. Age and mother's education were significantly associated with light PA, with older children, and those whose mother had less than college education engaging in fewer minutes of light PA. There were no associations between ethnic minority status and any PA intensity.

Comparisons across countries for the PA variables and sedentary behavior are summarized in Table 3. Least squares means (LS Means (95% CI)) are presented with adjustment for accelerometer wear time, age, gender, mother's education, ethnic minority (for country) or race/ethnicity (for regions within the U.S.), season, and BMI. Multiple regression yielded statistically significant intercountry differences for all the variables. Children from the U.S. were consistently lower than children in the European countries for all the PA variables and were higher for time spent in sedentary behavior, although not all differences were statistically significant. Among the three European countries, the PA variables tended to be highest in Finland, except for MVPA where Germany was slightly higher, and lowest in Sweden. There were no differences in PA variables between the U.S. and Germany. Children in the U.S. had significantly less TPA, light PA, MPA, and MVPA and more minutes of sedentary behavior than children in Finland. Children in the U.S. also had significantly less minutes of VPA than children in Sweden. Children in Finland had significantly more minutes of TPA and light PA and significantly fewer minutes of sedentary behavior than children in Sweden. All other comparisons between the European countries were not significantly different.

The findings for comparisons across the three U.S. regions are also presented in Table 3. Statistically significant interregion differences were observed for light PA, MPA, VPA, and MVPA. The PA variables were lower for children at the Southeast and Colorado study sites, than for those in Washington, except for light PA and VPA. For light PA, children in Colorado had significantly more minutes than children in Washington. For VPA, children in the Southeast region had fewer minutes than those in Washington.

## 4. Discussion

The major finding of this study was that 5-year-old children in the U.S. were observed to be less physically active, in general, than those in Finland, Germany, and Sweden. Time spent in MVPA was significantly lower in U.S. children than in those from Finland and Sweden, and this was observed in both unadjusted analyses and after adjustment for demographic characteristics and weight status. To our knowledge, this is the first cross-country comparison of PA in children as young as 5 years, and it is one of very few such studies to have used accelerometry as an objective measure of PA. In a comparison of PA among 9-11-year-old children from 12 countries, the highest levels of activity were found in Finnish children and U.S. children were the least active based on data from accelerometers [11]. Studies using self-reported measures of PA in children (11-15-year-olds) have described inconsistent findings [21, 22]. However, in a large-scale review of physical activity levels in 13-15-year-olds in 105 countries, Hallal et al. found that PA in U.S. adolescents was well below the pooled adjusted mean [23].

We found that PA levels differed in children across the three U.S. regions from which the sample was drawn. Physical activity was highest among children in the Northwest region (state of Washington) and lowest in the Southeast region (Georgia/Florida). Children in the Central region (state of Colorado) were midway between children from the other two regions. Similar patterns were found for all expressions of physical activity (i.e., MPA, VPA, MVPA, and TPA), and both with and without adjustment for demographic factors and weight status. These findings are unique in that this is the first study to report regional differences in physical activity levels in U.S. children as young as 5 years of age. However, the regional pattern observed in this study is similar to patterns previously reported in studies of older children. For example, the Trial of Activity in Adolescent Girls (TAAG) was a large-scale multicenter study that used accelerometry to assess PA in groups of girls recruited through study centers in six geographically distributed states [24]. Similar to the pattern observed in the present study, TAAG reported that physical activity levels were lowest in girls in the Southeast region (states of South Carolina and Louisiana) and highest in the West region (states of California and Arizona) [24]. The same pattern has been observed in population-based surveillance systems. The Youth Risk Behavior Survey (YRBS) assesses the prevalence of selected health behaviors in state-level samples of high school students [25]. In 2015, YRBS found that the prevalence of daily physical activity was lower in students residing in states in the Southeast region than other regions [25]. Likewise, the National Survey of Children's Health found that the prevalence of reporting no participation in vigorous intensity physical activity was highest in states in the Southeast region [26]. Multiple studies with varying methodologies have consistently found lower physical activity levels in children residing in the Southeast region of the U.S. as compared with other regions, and the present study shows that this pattern is evident even in children as young as 5 years of age. The underlying explanation for this pattern is not known, though several social and demographic factors may contribute. These include cultural factors related to race and ethnicity, poverty rate, and community supports for physical activity [27].

In addition to the differences in PA, our study found cross-country differences in the amount of sedentary behavior among the children. Minutes of sedentary behavior were highest among U.S. children and lowest in Finnish children. Within the U.S., the Southern states had the highest sedentary minutes per day, followed by Colorado, with Washington having the lowest (not significantly different). These differences are important because there is a growing body of evidence linking high levels of sedentary behavior to adverse health outcomes [28, 29]. Few studies have compared sedentary behavior estimates across countries and regions using objective measures and none in this age group. Previous studies in older age groups have shown that minutes of sedentary behavior are higher in the U.S. compared to other countries [11, 23] and are higher in the Southeast compared to other regions of the U.S. [24, 30]. The combination of low activity and high sedentary time in these populations is of concern and warrants targeting interventions to both increase PA and reduce sedentary time.

The strengths of this study include the use of an objective measure of PA in a large, multinational sample. In addition, data were collected year-round in all the countries, reducing the potential influence of seasonality on physical activity behaviors. It should be noted that the sites that participated in this study do not represent the whole of the geographic regions referenced (Europe or regions of the U.S.). The use of accelerometry to measure PA may result in underestimates of activity since they do not measure water or cycling activities well. Additionally, the use of accelerometers requires analysis decisions to be made regarding compliance, wear time, and cut-points that influence outcomes. Another limitation is that participants in this study were at an elevated risk for development of T1D, although children were excluded from the analysis if they were antibody positive at or before the time of the 5-year-old measurement. It is nonetheless possible that parental knowledge of this risk led to changes in children's behaviors, including their PA.

## 5. Conclusions

Previous studies have observed that adolescents and adults in the U.S. are less physically active than their counterparts in European countries, and the findings of the present study support the conclusion that this pattern extends to children as young as 5 years of age. Within the U.S., we observed that 5-year-old children in the Southeast region were less physically active, in general, than those in the West region. These differences persisted after adjustment for child-level characteristics, including gender, weight status, race/ethnicity, mother's education, and season of the year. Future studies should be designed to identify social and physical environmental factors and cultural characteristics that may explain why young children's PA levels differ across countries and across U.S. regions.

## Figures and Tables

**Table 1:** Participant characteristics.

		Total *n* = 1984	U.S. *n* = 826	Finland *n* = 368	Germany *n* = 85	Sweden *n* = 705	*p* value	Colorado *n* = 367	GA/FL *n* = 125	Wash. *n* = 334	*p* value
Accelerometer wear time, minutes/day		1049.5 (160.3)	1018.2 (174.8)^a^	1048.2 (153.1)^b^	1024.7 (139.0)^a,b^	1089.9 (138.3)	<0.0001	1084.3 (146.8)	1026.4 (154.1)	942.5 (180.6)	<0.0001

Child demographic characteristics, weight status, and season											
Age, months; mean (SD)		61.2 (1.5)	61.2 (1.4)^a^	61.5 (1.6)	62.9 (1.8)	61.0 (1.3)^a^	<0.0001	60.7 (1.4)	61.8 (1.5)	61.4 (1.4)	<0.0001
Body weight, kg; mean (SD)		19.7 (2.8)	19.3 (2.7)^a^	19.6 (2.8)^b^	19.7 (2.6)	20.1 (2.7)^a,b^	<0.001	18.9 (2.5)^a^	19.3 (2.3)^a,b^	19.7 (3.1)^b^	0.001
BMI, kg/m^2^; mean (SD)		15.8 (1.4)	15.7 (1.4)^a^	15.9 (1.4)^a,b^	15.3 (1.3)	16.0 (1.4)^b^	<0.001	15.6 (1.3)	15.7 (1.3)	15.9 (1.5)	0.10
Males, *N* (%)		1019 (51.4)	435 (52.7)	186 (50.5)	47 (55.3)	351 (49.8)	0.60	202 (55.0)	60 (48.0)	173 (51.8)	0.36
Race/ethnicity; *N* (%)	Hispanic							103 (28.1)	8 (6.4)^a^	23 (6.9)^a^	<.0001
	White, Non-Hispanic							256 (69.8)	107 (85.6)	276 (82.6)	
	African American, Non-Hispanic							2 (0.5)	4 (3.2)	5 (1.5)	
	Other							5 (1.4)	4 (3.2)	19 (5.7)	
	Missing/unknown							1 (0.3)	2 (1.6)	11 (3.3)	
Ethnic minority, yes; *N* (%)		271 (13.9)	211 (26.2)	10 (2.8)^a^	13 (15.3)	37 (5.3)^a^	<0.0001	121 3(33.2)	26 (21.0)^a^	64 (20.1)^a^	<0.001
Season; *N* (%)	Fall	465 (23.4)	173 (20.9)	94 (25.5)	23 (27.1)	175 (24.8)	0.05	84 (22.9)	19 (15.2)	70 (21.0)	.69
	Spring	524 (26.4)	208 (25.2)	85 (23.1)	23 (27.1)	208 (29.5)		91 (24.8)	34 (27.2)	83 (24.9)	
	Summer	506 (25.5)	234 (28.3)	115 (31.3)	17 (20.0)	140 (20.0)		98 (26.7)	37 (29.6)	99 (29.6)	
	Winter	489 (24.7)	211 (25.5)	74 (20.1)	22 (25.9)	182 (25.8)		94 (25.6)	35 (28.0)	82 (24.6)	

Family characteristics											
Mother's education; *N* (%)	Less than college grad	726 (36.9)	272 (33.1)^a^	113 (31.1)^a^	44 (51.8)^b^	297 (42.5)^b^	<0.0001	235 (64.6)^a^	97 (78.2)	218 (65.3)^a^	.01
	College grad or higher	1243 (63.1)	550 (66.9)	250 (68.9)	41 (48.2)	402 (57.5)		129 (35.4)	27 (21.8)	116 (34.7)	

BMI = body mass index; values with the same superscript are not significantly different.

**Table 2:** Results of regression analyses for PA intensity categories and demographic factors, BMI, and season of the year.

	Light estimate (95% CI)	MPA estimate (95% CI)	VPA estimate (95% CI)	MVPA estimate (95% CI)	Total PA estimate (95% CI)	Sedentary estimate (95% CI)
Intercept	248.0 (171.2, 324.7)^∗∗∗^	82.1 (15.84, 148.3)^∗^	11.7 (-7.55, 31.0)	93.8 (16.1, 171.5)^∗^	341.8 (231.3, 452.2)^∗∗∗^	-341.8 (-452.2, -231.3)^∗∗∗^
Wear time, min/day	0.19 (0.18, 0.20)^∗∗∗^	0.05 (0.04, 0.06)^∗∗∗^	0.01 (0.006, 0.01)^∗∗∗^	0.06 (0.05, 0.07)^∗∗∗^	0.25 (0.24, 0.27)^∗∗∗^	0.75 (0.73, 0.76)^∗∗∗^
Age, years	-1.21 (-2.40, -0.03)^∗^	0.02 (-1.0, 1.05)	0.01 (-0.29, 0.30)	0.03 (-1.17, 1.23)	-1.19 (-2.89, 0.52)	1.19 (-0.52, 2.89)
BMI	0.60 (-0.66, 1.87)	-0.30 (-1.39, 0.79)	-0.43 (-0.75, -0.12)^∗∗^	-0.73 (-2.01, 0.55)	-0.13 (-1.95, 1.69)	0.13 (-1.69, 1.95)
Sex, females vs. males	11.0 (7.45, 14.55)^∗∗∗^	-24.96 (-28.0, -21.9)^∗∗∗^	-3.48 (-4.37, -2.58)^∗∗∗^	-28.43 (-32.0, -24.8)^∗∗∗^	-17.4 (-22.5, -12.5)^∗∗∗^	17.4 (12.3, 22.5)^∗∗∗^
Mother's education, less than college vs. more than college	4.52 (0.78, 8.26)^∗^	-1.18 (-4.41, 2.05)	-0.64 (-1.58, 0.30)	-1.82 (-5.61, 1.96)	2.69 (-2.69, 8.01)	-2.69 (-8.01, 2.69)
Season						
Fall	2.20 (-2.87, 7.27)	0.40 (-4.00, 4.75)	1.19 (-.08, 2.46)	1.56 (-4.57, 6.70)	3.76 (-3.54, 11.1)	-3.76 (-11.06, 3.54)
Spring	0.66 (-4.27, 5.59)	5.12 (0.86, 9.37)^∗^	4.06 (2.83, 5.30)^∗∗∗^	9.18 (4.19, 14.17)^∗∗∗^	9.84 (2.74, 16.9)^∗^	-9.8 (-16.9, -2.75)^∗^
Summer	-0.02 (-5.01, 4.97)	-0.74 (-5.05, 3.57)	2.98 (1.72, 4.23)^∗∗∗^	2.24 (-2.82, 7.29)	2.21 (-4.97, 9.40)	-2.21 (-9.39, 4.97)
Winter	Reference	Reference	Reference	Reference	Reference	Reference
Ethnic minority, no vs. yes	-4.90 (-10.1, 0.27)	-0.38 (-4.84, 4.07)	0.76 (-0.54, 2.05)	0.37 (-4.86, 5.61)	-4.53 (-12.0, 2.91)	4.53 (-2.91, 11.97)

Note: each column represents a separate regression analysis for each expression of physical activity or sedentary behavior. Significant demographic predictors of PA or sedentary behavior are marked with asterisks. PA = physical activity, MPA = moderate physical activity, VPA = vigorous physical activity, MVPA = moderate-to-vigorous physical activity, ^∗^*p* < 0.05; ^∗∗^*p* < 0.01; ^∗∗∗^*p* < 0.001.

**Table 3:** Comparison of countries and regions within the U.S. for time spent in light, moderate (MPA), vigorous (VPA), moderate-to-vigorous (MVPA), total physical activity (TPA), and sedentary behavior (minutes per day).

Adjusted for wear time, age, gender, mother's education, season, BMI, and ethnic minority (*n* = 1911)^∗^			Adjusted for wear time, age, gender, mother's education, season, BMI, and race/ethnicity (*n*=802)^∗∗^		
	LS mean (95% CI)	*p* value		LS mean (95% CI)	*p* value
Light min/day		0.0001	Light min/day		0.05
U.S.	389.9 (386.8, 393.0)^a,b^		Colorado	384.0 (375.7, 392.3)^a^	
Finland	397.5 (392.7, 402.3)^c^		Georgia/Florida	378.4 (368.6, 388.2)^a,b^	
Germany	394.1 (385.2, 402.9)^a,c,d^		Washington	375.5 (367.5, 383.4)^b^	
Sweden	385.9 (382.1, 389.7)^b,d^				
MPA min/day		<0.0001	MPA min/day		<0.001
U.S.	121.0 (118.3, 123.6)^a^		Colorado	116.7 (109.7, 123.7)^a^	
Finland	130.6 (126.4, 134.7)^b,c^		Georgia/Florida	112.8 (104.6, 121.0)^a^	
Germany	131.4 (123.7, 139.0)^a,b,d^		Washington	125.5 (118.8, 132.2)	
Sweden	126.1 (122.8, 129.4)^c,d^				
VPA min/day		<0.0001	VPA min/day		0.02
U.S.	13.5 (12.6, 14.2)^a,b^		Colorado	13.5 (11.6, 15.5)^a,b^	
Finland	14.4 (13.2, 15.6)^a,c,d^		Georgia/Florida	12.4 (10.1, 14.7)^a^	
Germany	13.9 (11.7, 16.1)^b,c,e^		Washington	15.0 (13.2, 16.0)^b^	
Sweden	16.0 (15.1, 17.0)^d,e^				
MVPA min/day			MVPA min/day		<0.001
U.S.	134.4 (131.3, 137.5)^a^	<0.0001	Colorado	130.2 (122.0, 138.5)^a^	
Finland	145.0 (140.1, 149.9)^b,c^		Georgia/Florida	125.2 (115.6, 134.9)^a^	
Germany	145.3 (136.3, 154.3)^a,b,d^		Washington	140.5 (132.7, 148.4)	
Sweden	142.1 (138.3, 146.0)^c,d^				
Total PA min/day			Total PA min/day		0.13
U.S.	524.3 (519.9, 528.7)^a,b^	<0.0001	Colorado	514.2 (502.3, 526.2)	
Finland	542.5 (535.5, 549.4)^c^		Georgia/Florida	503.6 (489.6, 517.7)	
Germany	539.3 (526.5, 552.1)^a,c,d^		Washington	516.0 (504.6, 527.4)	
Sweden	528.0 (522.5, 533.5)^b,d^				
Sedentary min/day			Sedentary min/day		0.13
U.S.	526.4 (522.0, 530.8)^a,b^	<0.0001	Colorado	505.3 (493.0, 517.3)	
Finland	508.2 (501.3, 515.2)^c^		Georgia/Florida	515.9 (501.9, 529.9)	
Germany	511.4 (498.7, 524.2)^a,c,d^		Washington	503.6 (492.2, 514.9)	
Sweden	522.7 (517.2, 528.2)^b,d^				

PA = physical activity, MPA = moderate physical activity, VPA = vigorous physical activity, MVPA = moderate-to-vigorous physical activity, BMI = body mass index. ^∗^Countries with the same letter do not differ, after Bonferroni corrections. ^∗∗^Regions with the same letter do not differ, after Bonferroni corrections.

## Data Availability

The datasets generated and analyzed during the current study will be made available in the NIDDK Central Repository at https://www.niddkrepository.org/studies/teddy
