# Corrigendum to “Cross-Country Comparisons of Physical Activity and Sedentary Behavior Among 5-Year-Old Children”

**DOI:** 10.1155/ijpe/9818742

**Published:** 2025-08-13

K. L. McIver, R. R. Pate, M. Dowda, S. B. Johnson, J. Yang, M. Butterworth, X. Liu, “Cross-Country Comparisons of Physical Activity and Sedentary Behavior Among 5-Year-Old Children”, *International Journal of Pediatrics* 2020 (2020): 7912894, https://doi.org/10.1155/2020/7912894

In the article, the authors have identified errors in the Results section and in Table 1.

In the results section,

“Mother's education was different across the countries, with more mothers in the United States and Finland reporting education levels of college graduate or higher and more mothers in Germany and Switzerland reporting education levels less than college graduates.”

should read as follows:

“Mother's education was different across the countries, with more mothers in the United States and Finland reporting education levels of college graduate or higher and more mothers in Germany and Sweden reporting education levels less than college graduates.”

Additionally, incorrect values were included in the Family Characteristics section the Table 1, specifically in the Colorado, GA/FL, and Wash. columns. The correct Table 1 is shown below.

The authors apologize for these errors.

## Figures and Tables

**Table 1:** Participant characteristics.

		**Total (** **n** = 1984**)**	**United States (** **n** = 826**)**	**Finland (** **n** = 368**)**	**Germany (** **n** = 85**)**	**Sweden (** **n** = 705**)**	**p** ** value**	**Colorado (** **n** = 367**)**	**GA/FL (** **n** = 125**)**	**Wash. (** **n** = 334**)**	**p** ** value**
Accelerometer Wear time (min/day)		1049.5 (160.3)	1018.2 (174.8)^a^	1048.2 (153.1)^b^	1024.7 (139.0)^a,b^	1089.9 (138.3)	< 0.0001	1084.3 (146.8)	1026.4 (154.1)	942.5 (180.6)	< 0.0001

Child demographic characteristics, weight status, and season											
Age (months), mean (SD)		61.2 (1.5)	61.2 (1.4)^a^	61.5 (1.6)	62.9 (1.8)	61.0 (1.3)^a^	< 0.0001	60.7 (1.4)	61.8 (1.5)	61.4 (1.4)	< 0.0001
Body weight (kg), mean (SD)		19.7 (2.8)	19.3 (2.7)^a^	19.6 (2.8)^b^	19.7 (2.6)	20.1 (2.7)^a,b^	< 0.001	18.9 (2.5)^a^	19.3 (2.3)^a,b^	19.7 (3.1)^b^	0.001
BMI, (kg/m^2^), mean (SD)		15.8 (1.4)	15.7 (1.4)^a^	15.9 (1.4)^a,b^	15.3 (1.3)	16.0 (1.4)^b^	< 0.001	15.6 (1.3)	15.7 (1.3)	15.9 (1.5)	0.10
Males, *N* (%)		1019 (51.4)	435 (52.7)	186 (50.5)	47 (55.3)	351 (49.8)	0.60	202 (55.0)	60 (48.0)	173 (51.8)	0.36

Race/ethnicity, *N* (%)	Hispanic							103 (28.1)	8 (6.4)^a^	23 (6.9)^a^	< 0.0001
	White, non-Hispanic							256 (69.8)	107 (85.6)	276 (82.6)	
	African American, non-Hispanic							2 (0.5)	4 (3.2)	5 (1.5)	
	Other							5 (1.4)	4 (3.2)	19 (5.7)	
	Missing/unknown							1 (0.3)	2 (1.6)	11 (3.3)	

Ethnic minority, yes; *N* (%)		271 (13.9)	211 (26.2)	10 (2.8)^a^	13 (15.3)	37 (5.3)^a^	< 0.0001	121 (33.2)	26 (21.0)^a^	64 (20.1)^a^	< 0.001

Season, *N* (%)	Fall	465 (23.4)	173 (20.9)	94 (25.5)	23 (27.1)	175 (24.8)	0.05	84 (22.9)	19 (15.2)	70 (21.0)	0.69
	Spring	524 (26.4)	208 (25.2)	85 (23.1)	23 (27.1)	208 (29.5)		91 (24.8)	34 (27.2)	83 (24.9)	
	Summer	506 (25.5)	234 (28.3)	115 (31.3)	17 (20.0)	140 (20.0)		98 (26.7)	37 (29.6)	99 (29.6)	
	Winter	489 (24.7)	211 (25.5)	74 (20.1)	22 (25.9)	182 (25.8)		94 (25.6)	35 (28.0)	82 (24.6)	

Family characteristics											
Mother's education, *N* (%)	Less than college grad	726 (36.9)	272 (33.1)^a^	113 (31.1)^a^	44 (51.8)^b^	297 (42.5)^b^	< 0.0001	129^a^(35.4)	27 (21.8)	116^a^ (34.7)	0.01
	College grad or higher	1243 (63.1)	550 (66.9)	250 (68.9)	41 (48.2)	402 (57.5)		235 (64.6)	97 (78.2)	218 (65.3)	

*Note:* Values with the same superscript are not significantly different.

Abbreviation: BMI, body mass index.

